# Segmentation of 4D Flow MRI: Comparison between 3D Deep Learning and Velocity-Based Level Sets

**DOI:** 10.3390/jimaging9060123

**Published:** 2023-06-19

**Authors:** Armando Barrera-Naranjo, Diana M. Marin-Castrillon, Thomas Decourselle, Siyu Lin, Sarah Leclerc, Marie-Catherine Morgant, Chloé Bernard, Shirley De Oliveira, Arnaud Boucher, Benoit Presles, Olivier Bouchot, Jean-Joseph Christophe, Alain Lalande

**Affiliations:** 1 CASIS—Cardiac Simulation & Imaging Software, 21800 Quetigny, France; abarrera@casis.fr (A.B.-N.); tdecourselle@casis.fr (T.D.); sdoliveira@casis.fr (S.D.O.); jjchristophe@casis.fr (J.-J.C.); 2 IFTIM, ICMUB Laboratory, University of Burgundy, 21078 Dijon, France; dianamarin228@gmail.com (D.M.M.-C.); siyu.lin@u-bourgogne.fr (S.L.); sarah.leclerc@u-bourgogne.fr (S.L.); mariecatherine.morgant@chu-dijon.fr (M.-C.M.); chloe.bernard@chu-dijon.fr (C.B.); arnaud.boucher@u-bourgogne.fr (A.B.); benoit.presles@u-bourgogne.fr (B.P.); olivier.bouchot@chu-dijon.fr (O.B.); 3 Department of Cardio-Vascular and Thoracic Surgery, University Hospital of Dijon, 21078 Dijon, France; 4 Department of Medical Imaging, University Hospital of Dijon, 21078 Dijon, France

**Keywords:** segmentation, 4D flow MRI, deep learning, aorta

## Abstract

A thoracic aortic aneurysm is an abnormal dilatation of the aorta that can progress and lead to rupture. The decision to conduct surgery is made by considering the maximum diameter, but it is now well known that this metric alone is not completely reliable. The advent of 4D flow magnetic resonance imaging has allowed for the calculation of new biomarkers for the study of aortic diseases, such as wall shear stress. However, the calculation of these biomarkers requires the precise segmentation of the aorta during all phases of the cardiac cycle. The objective of this work was to compare two different methods for automatically segmenting the thoracic aorta in the systolic phase using 4D flow MRI. The first method is based on a level set framework and uses the velocity field in addition to 3D phase contrast magnetic resonance imaging. The second method is a U-Net-like approach that is only applied to magnitude images from 4D flow MRI. The used dataset was composed of 36 exams from different patients, with ground truth data for the systolic phase of the cardiac cycle. The comparison was performed based on selected metrics, such as the Dice similarity coefficient (DSC) and Hausdorf distance (HD), for the whole aorta and also three aortic regions. Wall shear stress was also assessed and the maximum wall shear stress values were used for comparison. The U-Net-based approach provided statistically better results for the 3D segmentation of the aorta, with a DSC of 0.92 ± 0.02 vs. 0.86 ± 0.5 and an HD of 21.49 ± 24.8 mm vs. 35.79 ± 31.33 mm for the whole aorta. The absolute difference between the wall shear stress and ground truth slightly favored the level set method, but not significantly (0.754 ± 1.07 Pa vs. 0.737 ± 0.79 Pa). The results showed that the deep learning-based method should be considered for the segmentation of all time steps in order to evaluate biomarkers based on 4D flow MRI.

## 1. Introduction

Aortic aneurysm is a pathological condition characterized by the permanent dilation of the aorta, which can progressively lead to aortic wall ruptures. Current guidelines for the diagnosis and prognosis of this disease are based on the maximum diameter of the aorta, expansion rate and sex [1,2]. However, these parameters (particularly the maximum diameter [3,4]) become insufficient in the presence of some tissue disorders, so other metrics must be considered in order to anticipate the evolution of aortic aneurysm. With improvements in time resolved three-directional phase contrast MRI acquisition, commonly known as 4D flow, new relevant hemodynamic biomarkers, such as wall shear stress (WSS) and vorticity, have become popular for the study of aortic diseases [5]. However, the computer-assisted analysis of these parameters requires the proper definition of the aortic wall during all phases of the cardiac cycle. The low contrast of vessel boundaries in the anatomical volume, as well as the low spatial resolution and large size of 4D flow images, make manual segmentation time-consuming and challenging. Moreover, the application of basic image segmentation algorithms to fully capture blood vessels is ineffective due to high signal intensity variations between the different aortic regions (thoracic, abdominal, etc.).

### 1.1. Objective

This study aimed to compare two different methods based on distinct concepts for automatically segmenting the thoracic aorta in the systolic phase using 4D flow MRI. The first method, developed by the CArdiac Simulation and Imaging Software (CASIS) Company and integrated in QIR-4D Research v1.0 software, is based on a level set framework. This method uses the velocity field within a specific time frame (in our case, the systolic phase), in addition to a generated PC-MRA. The second method is a U-Net-like convolutional neural network that only uses anatomical information from magnitude images, which can be extended to 4D segmentation. The objective of this study was to ascertain whether direct segmentation from magnitude images is feasible using deep learning as velocity information can affect convolution due to high variations in the signals present in phase images. The study also aimed to compare two methods based on well-known concepts from the literature. Another additional objective was to evaluate a deep learning method from this specific field using a scarce expertise dataset. This study involved 36 patients with thoracic aortic aneurysm and the comparison of the selected approaches was performed both globally and locally.

### 1.2. Related Work

Our study was conducted within the context of image segmentation. Image segmentation is a fundamental preliminary step in a lot of areas, such as parameter extraction, classification tasks and object recognition, although image segmentation is not always mandatory for the latter example [6]. In previous works, the segmentation of the aorta has been performed using advanced algorithms based on the tubular structures of vessels, which has obtained promising results [7,8] at the cost of defining and adjusting one or more parameters. The use of such semi-automatic algorithms has become standard and is commonly proposed for clinical and research applications, usually involving an initial model, the parameters of which are adapted to the specific patient case, or the definition of one or more seed points for front propagation algorithms [9].

On the other hand, methods for the automatic detection of the aorta have recently been proposed. Among them, atlas-based approaches can provide the fully automatic segmentation of one volume for a specific cardiac phase, which is then propagated through the cardiac cycle [10]. Although the overall results are encouraging for the analysis of hemodynamic parameters, these algorithms require large computational resources and are time-consuming for clinical analysis. In recent years, deep learning-based methods have gained popularity due to their capacity to obtain fully automatic segmentation in a short time, even outperforming current commercial solutions when comparing blood flow parameters [11] and achieving a Dice score of 0.94 in both 2D and 3D segmentation [12,13]. This makes deep learning a potential tool for implementation in clinical workflows, particularly for the management of 4D flow MRI [14,15,16]. The precise definition of the aorta during each phase of the cardiac cycle is necessary for the optimal generation of advanced hemodynamic markers. However, most of the segmentation methods proposed to date have sacrificed this need in order to generate single 3D images by combining 4D flow MRI frames to facilitate segmentation by obtaining a better contrast between the aorta and the background. Among the types of 3D images generated using 4D flow MRI are maximum intensity projection images (tMIPs) [17] and 3D phase contrast magnetic resonance angiography (PCMRA) [14,18,19]. With the lack of information on the dynamic position of the aorta, it is difficult to extend these methods to the segmentation of the aorta throughout the entire cardiac cycle (i.e., 4D segmentation). The comparison of 4D flow segmentation has been carried out before using similarly fully automatic velocity- and CNN-based methods in the cerebrovascular region [20]. Within this framework, our study aimed to compare two advanced approaches for automatically segmenting the aorta using 4D flow MRI.

## 2. Materials and Methods

### 2.1. Study Population

The study was approved by the French national ethic committee (Project 2018-A02010-55, approved by the French “Comite de Protection des Personnes”) and was registered on ClinicalTrials.gov with the number NCT03817008. The inclusion criteria for patients in this project were an age higher than 18 years old and a planned ascending aorta replacement with a graft. The exclusion criteria were prior cardiac surgery and any contraindication to performing cardiac MRI. The study group was composed of 26 males and 10 females, with an average age of 60 ± 15 years. Of these participants, 18 had a bicuspid aortic valve (BAV), while the other 18 had a tricuspid aortic valve (TAV). Within this group, 18 patients received beta blocker treatment, 2 were on antiarrhythmics and 1 was prescribed anticoagulants. Blood pressure was managed with ACE inhibitors in 10 patients and with angiotensin II receptor blockers (ARA II) in 6 patients. Additionally, seven patients received statins for managing cholesterol levels. Moreover, 3 patients were diabetic, 8 were classified as obese and 18 had dyslipidemia. Hypertension was observed in 22 patients, 6 were current smokers and 7 had quit smoking.

### 2.2. 4D Flow MRI Acquisition Settings

In this study, 36 patients with a thoracic aneurysm in the ascending aorta were included. For each patient, 4D flow MRI was performed using a 3 Tesla Siemens scanner (Skyra, Siemens Healthineers, Erlangen, Germany) after an injection of a gadolinium-based contrast agent. The sequence was an updated version of the WIP number 785, developed by Siemens, with retrospective gating and sparse sampling but without compressed sensing. Four-dimensional flow is the result of combining three-dimensional spatial encoding, velocity encoding in three directions and cine acquisition (3D+time). Consequently, the resulting image set comprises a magnitude volume and three volumes encoding phase differences in the x, y and z directions of space. In the reconstruction process, the Maxwell coefficients were corrected to minimize the impact of the accompanying gradient field. Additionally, non-uniform intensity correction and 2D distortion correction were also applied.

In total, 25 frames from throughout the cardiac cycle were acquired with a spatial resolution of 2 × 2 × 2 mm${}^{3}$ and a temporal resolution of between 24 and 52 ms, according to the cardiac rhythm of the patient. The field of view (FOV) was 262–350 mm × 350 mm and the velocity encoding was set in the range of 200 to 550 cm/s, depending on the patient (with the particularly extreme case of a patient with acute aortic stenosis and a bicuspid valve, for which the velocity encoding was set to 800 cm/s). Having uncommon cases where the velocity encoded speed was unconventional allowed us to challenge the methods using abnormally high speeds. Four-dimensional flow MRI was acquired during free-breathing with ECG gating using an echo navigator to manage diaphragmatic movement. The duration of the scan was 10 to 15 min, depending on the patient.

### 2.3. Ground Truth

The ground truth segmentations were generated by merging manual segmentations from two experienced cardiac image analysts. First, each observer manually delineated the aorta of each patient in the systolic phase using magnitude images. The magnitude image volumes corresponding to the systolic phase were identified by considering the maximum average velocity measured in a plane perpendicular to the ascending aorta. Later, to merge the manual segmentations, the Simultaneous Truth And Performance Level Estimation (STAPLE) algorithm was used to calculate a probabilistic estimate of the true segmentation [21]. ITK-SNAP software v4.0, University of Pennsylvania, Philadelphia, PA, USA) was used to perform the manual contouring. The brachiocephalic artery, left common carotid artery and left subclavian artery were excluded because our work only focused on the segmentation of the aorta, so other vessels were not considered.

### 2.4. Pre-Processing and Post-Processing

The pre-processing of the dataset was performed using QIR-4D Research software, consisting of phase offset correction, which was applied to the three phase contrast volumes following the volumetric approach of Craiem et al. [22]. Low-magnitude voxels were discarded according to a pre-set threshold, defined as the 15th percentile of the magnitude volume. For each phase direction, stationary tissues were selected when their temporal standard deviation was lower than the median temporal standard deviation. Then, a third degree surface was fitted using the ordinary least squares method, using the position and mean velocity of each stationary tissue sample to recover an offset volume. Finally, the offset was subtracted from the original phase volume. To train the deep learning model, magnitude images were normalized between 0 and 1.

As post-processing, using the Insight Toolkit (ITK-5.2.0) library, the largest object was retained as the final segmentation of the aorta. Moreover, a morphological opening was applied using a ball structuring element with a radius of 3 mm to smooth the segmentation.

### 2.5. Segmentation with Level Sets

**Figure 1:**
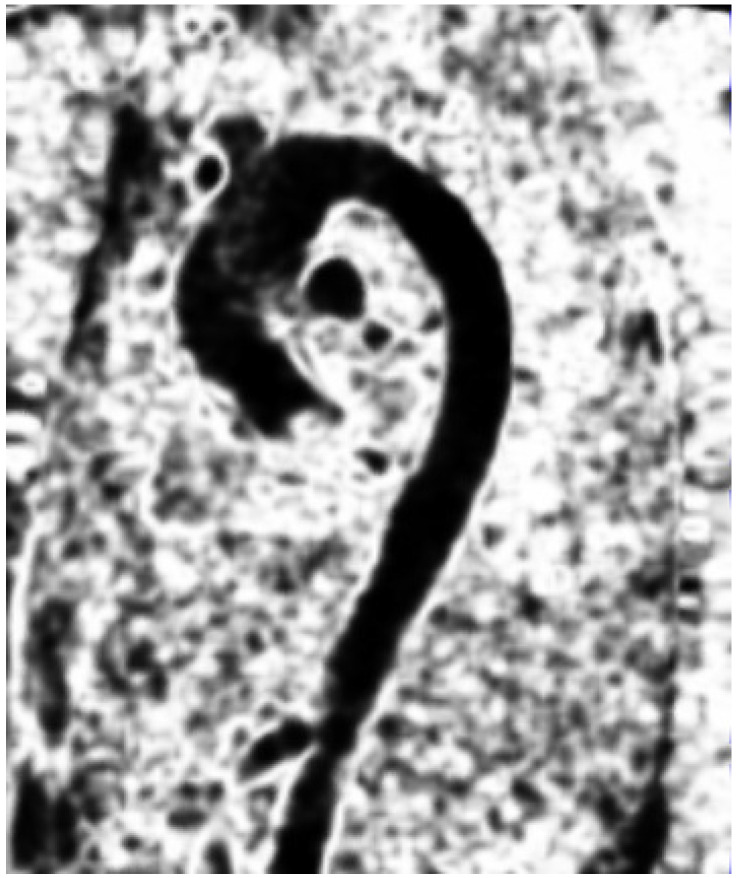
The discontinuity field on a sagittal 4D flow image during the systolic phase.

In contrast to the anatomical information provided by magnitude images, blood flow information contained in the velocity field *v* provides a reliable representation of a vessel’s inner walls. More precisely, and as proposed by Solem et al. [23], we could consider the density matrix field as follows:

(1) $${\bar{M}}_{v}=G_{\sigma }*\left[\begin{array}{ccc} v_{x}^{2} & v_{x}v_{y} & v_{x}v_{z}\\ v_{x}v_{y} & v_{y}^{2} & v_{y}v_{z}\\ v_{x}v_{z} & v_{y}v_{z} & v_{z}^{2} \end{array}\right]$$

where $G_{\sigma }$ is a Gaussian weight function and $v_{i}$ is the velocity in the *i* direction. The eigenvalues of ${\bar{M}}_{v}$ represent the magnitude of the three most dominant directions of flow for a given region of the volume. Given that a vessel’s walls are regions where two dominant directions are present during flow jets (i.e., the direction of the flow and the direction perpendicular to the wall), the following discontinuity function *R* was introduced:

(2) $$R=\frac{4\lambda_{1}\lambda_{2}}{{\left(\lambda_{1}+\lambda_{2}\right)}^{2}}$$

ranging from R=0 (where a region only had one dominant direction) up to R=1 (where a region had two or more dominant directions). Figure 1 presents the results of this discontinuity function for a 4D flow velocity field.

The level set framework was then used to capture the interfaces by minimizing the following equation:

(3) E(Γ)=∫χ(v)dx+∫R(x)dx

where χ(v) translates into the Heaviside function $\chi \left(v\right)=\chi \left(|v|\right)=H\left(|v|-\delta \right)=H_{\delta }\left(|v|\right)$ with $\delta \in R_{+}$ and *H*, as follows:

(4) $$H\left(x\right)=\left\{\begin{array}{cc} 1 & x>\epsilon ,\\ 0 & x<-\epsilon ,\\ \frac{1}{2}\left(1+\frac{x}{\epsilon }+\frac{1}{\pi }\sin\frac{\pi x}{\epsilon }\right) & |x|<\epsilon . \end{array}\right.$$

Hence, *R* and χ were used as the propagation terms in the level set equation from [24] to be solved by the following gradient descent:

(5) $$\phi_{t}=\left(-\chi +R\right)\left|\nabla \phi \right|$$

The implementation of this algorithm was carried out in QIR-4D Research software. The parameters δ and ϵ for the χ function were set between 275 and 0.5, respectively, in relation to the noise found in the dataset, with a maximum of 1000 iterations. The systolic phase was chosen as the time frame because it is when the velocity is at its peak in the volume during the cardiac cycle. PC-MRA was used to exclude regions with noise, giving strong discontinuity at the vessel boundaries. The initial front of the level set ϕ(x(t),t=0) was a thresholded synthetic angiography that was generated by combining both the magnitude and phase volumes, which was inspired by Bustamante et al. [25] (Equation (6)).

(6) $$MRA=\frac{1}{N}\sum_{n=1}^{N}m\left(n\right)*{\left(v_{x}^{2}\left(n\right)+v_{y}^{2}\left(n\right)+v_{z}^{2}\left(n\right)\right)}^{\gamma }$$

where *N* is the number of time frames in the cardiac cycle, *m* is the magnitude volume and γ is set empirically to 0.2, which produced satisfactory results in terms of enhancing regions of low velocity.

### 2.6. Segmentation Using Deep Learning

**Figure 2:**
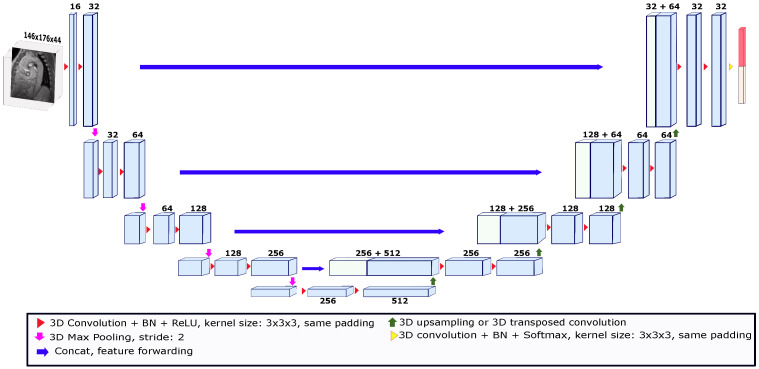
The 3D U-Net architecture used for the deep learning-based segmentation. The architecture was trained using the leave-one-patient-out strategy. The input image for each patient was a volume taken from a magnitude image of the systolic phase. The size of the input volume was set to 146 × 176 × 44. The numbers above the blocks indicate the number of feature maps.

Our deep learning segmentation algorithm is an end-to-end method where the model automatically learns image features to give each voxel a probability of belonging to the object of interest. Deep learning models for segmentation are usually convolutional (called convolutional neural networks or CNNs) [26]. One of the most widely known models used in the field of medical image segmentation is called U-Net. U-Net is a convolutional network that has been used for different medical image segmentation tasks in both 2D and 3D [27,28]. U-Net-like networks consist of two paths called encoders and decoders. The encoder path is made up of convolutional and pooling layers that spatially contract images while capturing their contextual information. The decoder path allows for precise location reconstruction by expanding the output to recover a full-resolution segmentation probability map. In this study, we used the 3D U-Net depicted in Figure 2 [29]. This U-Net consisted of a four-level encoder–decoder structure with skip connections to enable the preservation of spatial information throughout the network. The skip connections established direct pathways between corresponding encoder and decoder levels, facilitating the transfer of high-resolution features. This allowed the U-Net network to effectively capture fine details and local context while maintaining global information. The architecture was composed of convolution kernels of 3 × 3 × 3 voxels and batch normalization (BN) was employed after each layer to stabilize the learning process and improve performance. After each BN process, the rectified linear unit (ReLU) activation function was applied. Moreover, the expansion in the decoder path was performed using upsampling operations to reduce the number of parameters compared to up-convolutions. To feed the network, the images were cropped or padded based on the median size of the *x* and *y* axes and the maximum size of the *z* axis across the images in the dataset. Thus, the size of the images used to feed the model was set to 146 ×176 × 44 voxels. To train the U-Net model, a validation strategy known as leave-one-patient-out was used. This strategy is a variation of cross-validation, in which the k-folds number is equal to the number of patients in the dataset. Thus, for each patient used as a test, the images of 35 patients were used for training. The models were trained for 850 epochs with an initial learning rate of 0.01, which was reduced by a factor of 10 when the validation loss stopped improving. The deep learning model was implemented in PyTorch 1.9.0. The U-Net segmentation tests were run on a computational cluster with an NVIDIA Tesla V100 GPU.

### 2.7. Evaluation

**Figure 3:**
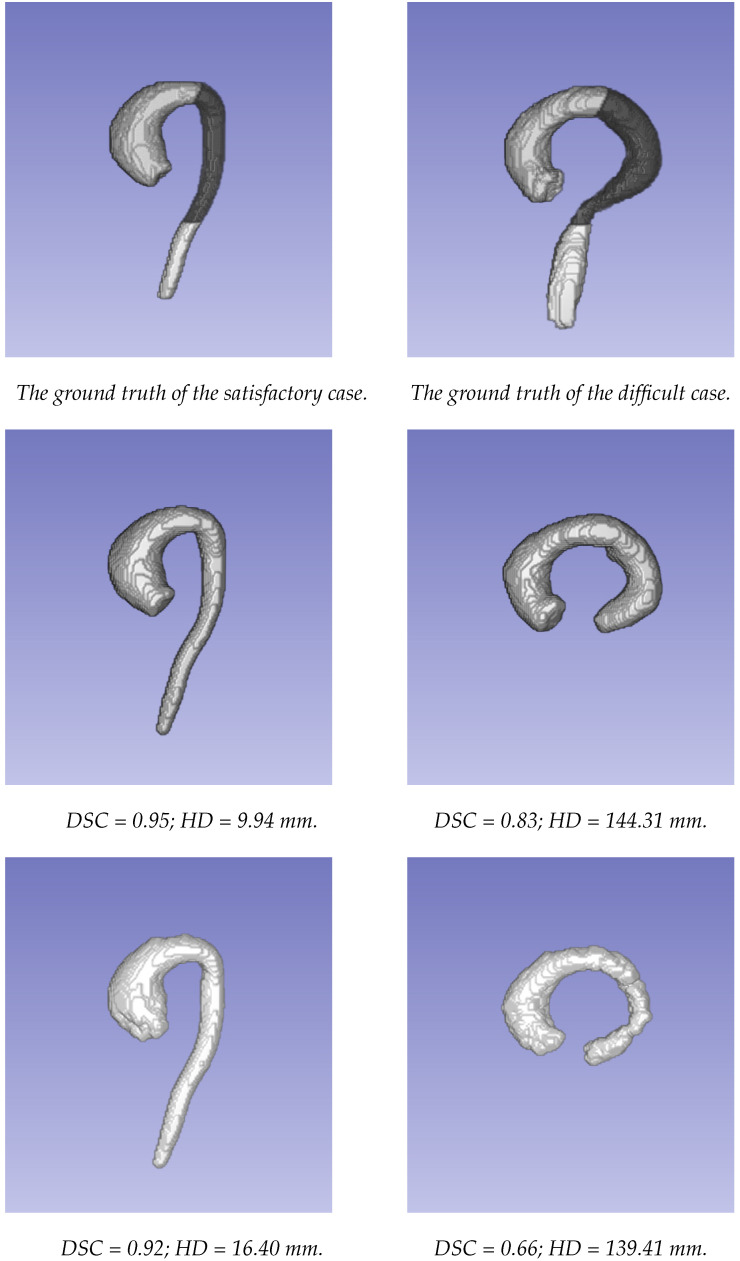
The examples of the segmentations obtained using the two approaches. The first column presents a case where both methods provided satisfactory results. The second column presents a case where both methods provided poor results. From top to bottom: ground truth data; segmentation using the U-Net network; segmentation based on the level set approach.

The segmentations obtained using the two approaches were compared to the ground truth segmentations generated using the STAPLE method. The comparison of the two approaches was first carried out for the whole aorta, from the valsalva sinus to the abdominal aorta (where there was an important decrease in signal because it was far from the center of the coil). To evaluate the behavior of each approach according to the localization, the aorta was divided into three parts: the ascending aorta, including the aortic arch; the descending thoracic aorta until the diaphragm (the dark part of the aorta in Figure 3); and the abdominal aorta (more specifically, only the visible part after the diaphragm, corresponding to the bright part of the aorta in Figure 3).

The overlap between the segmentations obtained using the two methods was evaluated using the Dice similarity coefficient (DSC) to obtain information on the global and local segmentation performance (Equation (7)) and the Hausdorff distance (HD) to evaluate the maximum distance between the two segmentations (Equation (8)).

(7) $$DSC\left(A,B\right)=2*\frac{A⋂B}{\left|A\right|+\left|B\right|}$$

(8) HD(A,B)=max(h(A,B),h(B,A))

where

(9) $$h\left(A,B\right)=\underset{a\in A}{max}\;\underset{b\in B}{min}∥a-b∥$$

If we consider two finite set of points $A=[a_{1},\ldots a_{p}]$ and $B=[b_{1},\ldots b_{p}]$, h(A,B) is a one-sided HD and $∥\cdot ∥$ is a measure of distance as the Euclidean norm. Thus, the function h(A,B) identifies the point *a* closest to any point of *B*. Then, it measures the distance from *a* to its nearest neighbor in *B* [30].

As wall shear stress directly benefits from the accurate computation of a vessel’s boundaries, it was assessed using QIR-4D Research software. First, meshes from both segmentation approaches and ground truth data were generated using a marching cubes algorithm. Then, for each point of the meshes, two additional points were placed along the inward normal vector of the point, separated from each other by a voxel’s size. The magnitude of the velocity tangential to the wall at these three points was used to perform a parabolic fitting, following the method described by Osinnski et al. [31]. Assuming there was no velocity at the boundary (no-slip boundary condition), the velocity profile’s tangent at the vessel wall was multiplied by the blood’s viscosity, as follows:

(10) $$\tau_{w}=\mu {\frac{\partial v}{\partial y}}_{y=0}$$

The blood viscosity μ was set to 3.2 cP. To compare both methods, the absolute difference in maximum wall shear stress between the automatic and manual methods was evaluated.

Moreover, for each parameter, a paired sample *t*-test was performed in order to determine whether the difference between the two methods was statistically significant.

## 3. Results

Table 1 summarizes the results of the two methods considering the Dice index, the Hausdorff distance and the absolute difference in wall shear stress for the whole aorta and the three selected regions. Regarding the Dice index and Hausdorff distance, the U-Net approach provided better results for every part of the aorta and the differences between the methods were statistically significant, except for the HD for the descending aorta. On another hand, the quantification of the maximum wall shear stress showed globally comparable results, slightly favoring the level set method, with the exception of the descending region. The level set method is based on velocity discontinuity, so it is possible that it succeeded at capturing regions of high velocity near the walls, despite underperforming at recovering the vessel walls.

Figure 3 displays the segmentations obtained using the two different approaches for an example of a patient with satisfactory results. The performance in the thoracic aorta was high. The highest HD for both methods was located at the end of the abdominal aorta. This region was challenging due to the drop in signal during acquisition. Furthermore, it could be noted on this patient that the discrepancies at the level of the valve were greater with the level set-based method. On the other hand, the lowest performance was observed in a patient with an atypical case, in which the image quality was highly affected in the abdominal area due to the unconventional orientation of the aorta with respect to the image acquisition directions.

Moreover, in general, the performance of the level set-based method was lower when the encoded velocity was high, corresponding to the high speed of the flow inside the aorta. Indeed, in this case, the amplitude of the velocity (and, by extension, when there were very high velocities) seemed not to be well managed by the level set method.

## 4. Discussion

The main objective of our work was to compare two distinct approaches for the segmentation of the aorta using 4D flow MRI. The selected methods outlined the main approaches used in the literature and reflected two different strategies. The method based on level sets used information from both magnitude and phase images but only considered one static phase of the cardiac cycle, as the diastolic phase does not provide enough velocity information for the discontinuity field. The method based on deep learning only considered magnitude images but could be extended to the whole cardiac cycle. To summarize the advantages of each, the level set method benefits from all data (magnitude and phase images) while the deep learning method only uses anatomical information from magnitude images but can be extended to any phase of the cardiac cycle. A second dimension of this work was to compare non-deep learning and deep learning methods on a scarce and noisy dataset that could be seen as representative of challenging datasets and, by extension, deep learning tasks within medical imaging.

One of the main results observed was the high potential for the use of deep learning in the segmentation of the aorta and the possibility of future extension to 4D segmentation. This result is important since the accurate calculation of biomechanic parameters, such as wall shear stress, requires FSI modeling using knowledge of the shape of the aorta for each time step [32]. Subsequently, only considering one time step is a major limitation for the assessment of certain pathologies, such aortic aneurysm.

As a second key point of this work, we corroborated the fact that the deep learning-based method outperformed a method often used for this kind of problem, even on a small dataset with a lot of anatomical variations. Despite the small dataset, the U-Net model showed a better aortic segmentation performance compared to the state-of-the-art level set method. This could have been related to the potential of the model architecture. For instance, we could cite the ability of U-Net to preserve and reconstruct spatial information from skip connections, which, in this case, also benefited from the standard position and orientation of the type of tissue to be segmented (i.e., the aorta). Moreover, it has been seen in related work that even 4D models have been successfully trained using small datasets (around 60 samples) [33]. In a previous work [16], we tested the same model using several frames from each patient, which could be seen as data augmentation. With this strategy, we went from having 35 to 175 training examples by using the leave-one-patient-out strategy. The average segmentation results from the data augmentation strategy were similar to those obtained in this paper.

In this study, the two approaches were compared using the DSC and HD. We did not consider other metrics, such as the Jaccard index or IoU (intersection over union), because we considered them to be redundant due to the DSC. Nevertheless, another parameter that could have been considered was the maximum diameter obtained from each model, but we chose not to consider this because the maximum diameter only depicts local information. The wall shear stress metric was considered to complement the comparison between the two methods, offering insights into the quantification stage. However, the comparison remained difficult and the variability of the biomarkers in this study ranged from 0.736 Pa to 12.293 Pa. Although WSS was calculated for each region using the maximum value throughout the cardiac cycle, perhaps measuring and comparing the means and standard deviations of each cardiac phase, as calculated by Stalder et al. [34] between 2D and 3D WSS, would provide information that is more exhaustive. Nonetheless, as the temporal resolution was different for each patient, the feasibility of such comparison remains uncertain. Further work on computation could also be carried out by separately calculating the axial and circumferential WSS instead of only the magnitude of the tangential velocity vector.

Although the results from the U-Net-based approach were encouraging, more data are needed to train the network in order to produce results that are always acceptable in clinical practice. Moreover, the data must come from different MR scanners from different manufacturers in order to support a robust software solution. Indeed, in our work, the data only came from one manufacturer, so we cannot be sure that the results would be similar using data from other manufacturers, even when the MRI acquisition protocols are comparable. Undoubtedly, the main drawback of this approach is access to data. Indeed, the 4D flow MRI of the thoracic aorta is not the most common MRI exam and including more exams would take time, even if a multi-site organization were designed. In addition to data collection, the annotation is a tedious and long process that increases the difficulty of obtaining the data. Moreover, the inter-observer variability could be high due to the noisy aspects of the images and reliable ground truth data require segmentations from several experts. On the other hand, the advantage of the level set-based method is that it does not require additional data to be improved. However, one drawback of this approach is the inability to manage cases with high velocity, which must be addressed in future work.

One constraint of this work was the restricted testing methods, so more methods should be considered in future work. Moreover, the U-Net network must be evaluated for all cardiac phases. This could reinforce the message that it is possible to segment the aorta using 4D flow MRI during all time steps using a deep learning-based method, even with a scarce dataset. Finally, the brachiocephalic artery, left common carotid artery and left subclavian artery were not included in the segmentation task and must be considered in an updated version of this work because FSI modeling could require the localization of these vessels.

## 5. Conclusions

The evaluation of aortic aneurysm is traditionally based on criteria that are not totally robust, such as the aortic diameter. Other biomechanical parameters should be considered in addition to the criteria in the guidelines, some of which could be obtained using 4D flow MRI, such as wall shear stress or the vorticity of the flow. As a part of this process, the automatic segmentation of the aorta is mandatory. In this work, we showed that the 3D segmentation of the aorta in using 4D flow MRI is feasible using only magnitude images thanks to the U-Net-based approach. Compared to the velocity-based level set method, the U-Net approach worked better. Our results demonstrated the strong potential of the use of deep learning to provide reliable segmentation results.

## Figures and Tables

**Table 1:** The comparison between the manual and automatic segmentations obtained using the U-Net- and level set-based approaches. The best results are highlighted in bold.

Metric	Region	U-Net	Level Set	*p*-Value
DSC	Whole Aorta	**0.92** ± **0.02**	0.86 ± 0.05	<$10^{-5}$
	Ascending Aorta	**0.93** ± **0.02**	0.88 ± 0.02	<$10^{-5}$
	Descending Aorta	**0.93** ± **0.02**	0.85 ± 0.08	<$10^{-5}$
	Abdominal Aorta	**0.84** ± **0.09**	0.72 ± 0.22	0.005
HD (mm)	Whole Aorta	**21.49** ± **24.8**	35.79 ± 31.33	0.002
	Ascending Aorta	**9.63** ± **3.4**	12.39 ± 4.84	0.013
	Descending Aorta	**5.97** ± **6.39**	7.11 ± 3.99	0.063
	Abdominal Aorta	**16.38** ± **13.6**	30.98 ± 27.31	0.002
Max WSS Absolute Difference (Pa)	Whole Aorta	0.754 ± 1.07	**0.737** ± **0.79**	0.907
	Ascending Aorta	0.743 ± 1.08	**0.742** ± **0.79**	0.992
	Descending Aorta	**0.116** ± **0.14**	0.133 ± 0.11	0.526
	Abdominal Aorta	0.155 ± 0.26	**0.112** ± **0.11**	0.354

## Data Availability

Belonging to the University Hospital of Dijon (France), data cannot be shared publicly without the permission of this institution. However, they are available upon request.
